# Antibody titres decline 3-month post-vaccination with BNT162b2

**DOI:** 10.1080/22221751.2021.1953403

**Published:** 2021-07-22

**Authors:** Julien Favresse, Jean-Louis Bayart, François Mullier, Marc Elsen, Christine Eucher, Sandrine Van Eeckhoudt, Tatiana Roy, Gregoire Wieers, Christine Laurent, Jean-Michel Dogné, Mélanie Closset, Jonathan Douxfils

**Affiliations:** a Department of Laboratory Medicine, Clinique St-Luc Bouge, Namur, Belgium; b Department of Pharmacy, Namur Research Institute for LIfe Sciences, University of Namur, Namur, Belgium; c Department of Laboratory Medicine, Clinique St-Pierre, Ottignies, Belgium; d Department of Laboratory Medicine, Université catholique de Louvain, CHU UCL Namur, Namur, Belgium; e Department of Internal Medicine, Clinique St-Luc Bouge, Namur, Belgium; f Department of Internal Medicine, Clinique St-Pierre, Ottignies, Belgium; g Department of Internal Medicine, Université catholique de Louvain, CHU UCL Namur, Namur, Belgium; h Qualiblood sa, Namur, Belgium

**Keywords:** COVID-19, SARS-CoV-2, mRNA vaccine, BNT162b2, antibody response

## Abstract

Several studies reported on the humoral response in subjects having received the BNT162b2 mRNA COVID-19 vaccine. However, data on the kinetics of antibodies 3 months post-vaccination are currently lacking and are important to drive the future vaccination strategy. The CRO-VAX HCP study is an ongoing multicentre, prospective and interventional study designed to assess the antibody response in a population of healthcare professionals who had received two doses of the BNT162b2 mRNA COVID-19 vaccine. Two hundred individuals underwent a blood drawn within 2 days before the first vaccine dose. One-hundred and forty-two persons (71%) were categorized as seronegative at baseline while 58 (29%) were seropositive. Samples were then collected after 14, 28, 42, 56, and 90 days. Antibodies against the SARS-CoV-2 nucleocapsid and the receptor binding domain of the S1 subunit of the spike protein were measured in all individuals at different time points. Using a one-compartment kinetics model, the time to maximum concentration was estimated at 36 ± 3 days after the first dose and the estimated half-life of antibodies was 55 days (95% CI: 37–107 days) in seronegative participants. In seropositive participants, the time to maximum concentration was estimated at 24 ± 4 days and the estimated half-life was 80 days (95% CI: 46–303 days). The antibody response was higher in seropositive compared to seronegative participants. In both seropositive and seronegative subjects, a significant antibody decline was observed at 3 months compared to the peak response. Nevertheless, the humoral response remained robust in all participants.

## Introduction

The efficacy and safety of the two-dose regimen of BNT162b2 mRNA COVID-19 vaccine (Pfizer-BioNTech, Mainz, Germany) has been proved and led to its approval by several regulatory authorities in late December 2020 [1]. Several studies have reported on the humoral response in vaccinated subjects but the results were only available after one administration of the vaccine and if the response after the second dose was evaluated, the follow-up of the participants was limited, i.e. below 3 months [2–6]. Therefore, a longer follow-up period is needed to assess the antibody kinetics in individuals after a two-dose regimen of BNT162b2. These data are important, especially since the question about a third dose has been raised by the pharmaceutical industries which will led to important societal, logistical and economical consequences.

## Material and methods

The CRO-VAX HCP study is an ongoing multicenter, prospective and interventional study designed to assess the antibody response in a population of healthcare professionals having received two doses of the BNT162b2 mRNA COVID-19 vaccine (Comirnaty®), as previously described in details [3]. All participants provided informed consent prior to collection of data and specimens. The study was approved by a central ethical committee (approval number: 2020-006149-21). Participants received the first vaccine dose from January 18, 2021, to February 17, 2021. The second dose was administered 21 days after the first one. All volunteers underwent blood drawn within 2 days before the first vaccine dose. Samples were then collected after 14, 28, 42, 56, and 90 days. Blood samplings performed earlier or later compared to the expected blood times collection were allowed (10% variation; i.e. 90 days ± 4.5 days). In this interim report, data from a total of 200 participants were available after three months.

Antibodies against the SARS-CoV-2 nucleocapsid (anti-NCP; Elecsys Anti-SARS-CoV-2 NCP total qualitative ECLIA, Roche Diagnostics, Machelen, Belgium) and the receptor binding domain of the S1 subunit of the spike protein (anti-S; Elecsys anti-SARS-CoV-2 spike total quantitative ECLIA, Roche Diagnostics) were measured at each time point. Results above 0.8 U/mL (manufacturer’s cut-off) or 0.165 COI (cut-off index; as found previously) for anti-S and anti-NCP antibodies were considered positives [7].

Means and 95% confidence intervals (95% CI) were used to describe the data. The between-group difference of antibody titres were tested using a Tukey multiple comparison test. A multiple testing correction was applied in the multiple group comparision. A one-compartment modelling was used to describe the kinetics of the antibody response in seropositive and seronegative subjects, assuming a steady decay rate over time. The half-life was obtained from the one-compartment modelling which permitted the calculation of the elimination rate of the antibody response. Statistical analyses were performed using GraphPad Prism 9.0.1 (GraphPad Software) and JMP Pro 16.0.0 (SAS Institute Inc., South Carolina, United States). *P*-value < 0.05 was considered significant.

## Results

**Figure 1.**
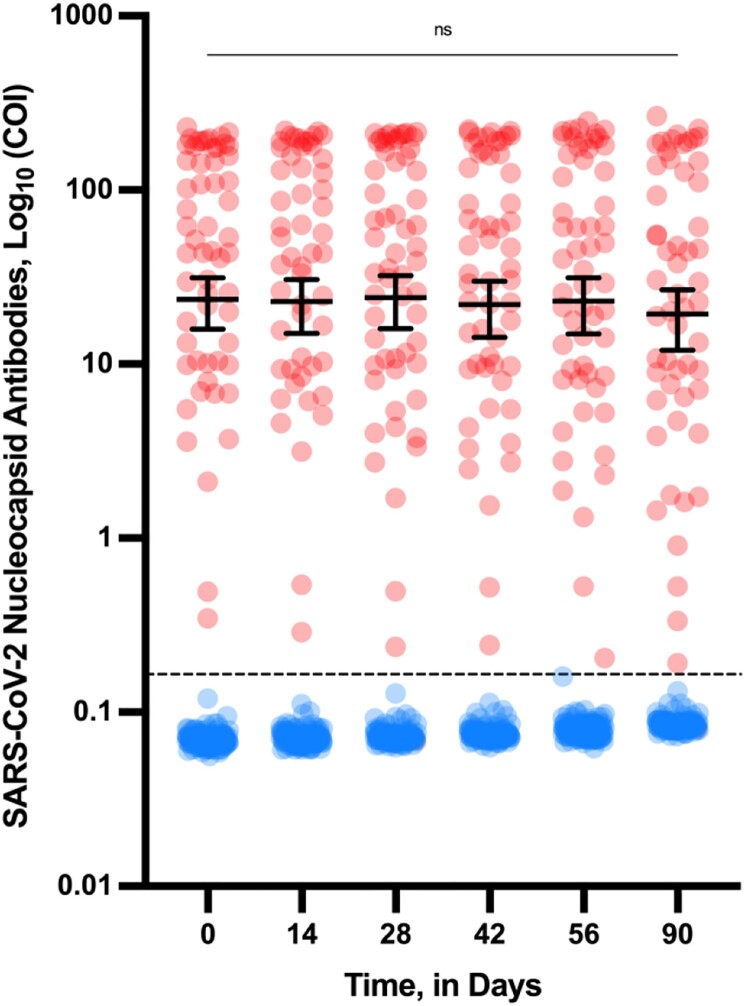
Evolution of SARS-CoV-2 nucleocapsid antibodies (COI) in seronegative (blue) and seropositive individuals (red) according to the time since the first vaccine dose administration. Means with 95% confidence intervals (log_10_) are shown. The black dotted line corresponds to the positivity cut-off (i.e. 0.165 COI). ns = non significant differences between timepoints (*P* < 0.05).

In this cohort, 77.5% (*n* = 155) were females (mean age = 43 years; range, 23–66 years) and 22.5% (*n* = 45) were males (mean age = 41 years; range, 24–64 years). One-hundred and forty-two persons (71%) were categorized as seronegative at baseline while 58 (29%) were seropositive (i.e. subjects having levels of anti-NCP and anti-S antibodies at baseline above the positivity cut-off). Anti-NCP antibodies remained stable in seropositive participants (Figure 1). In the seronegative group, no participant developed anti-NCP antibodies. None of the previously infected participants required hospitalization at the time of SARS-CoV-2 infection.

**Figure 2.**
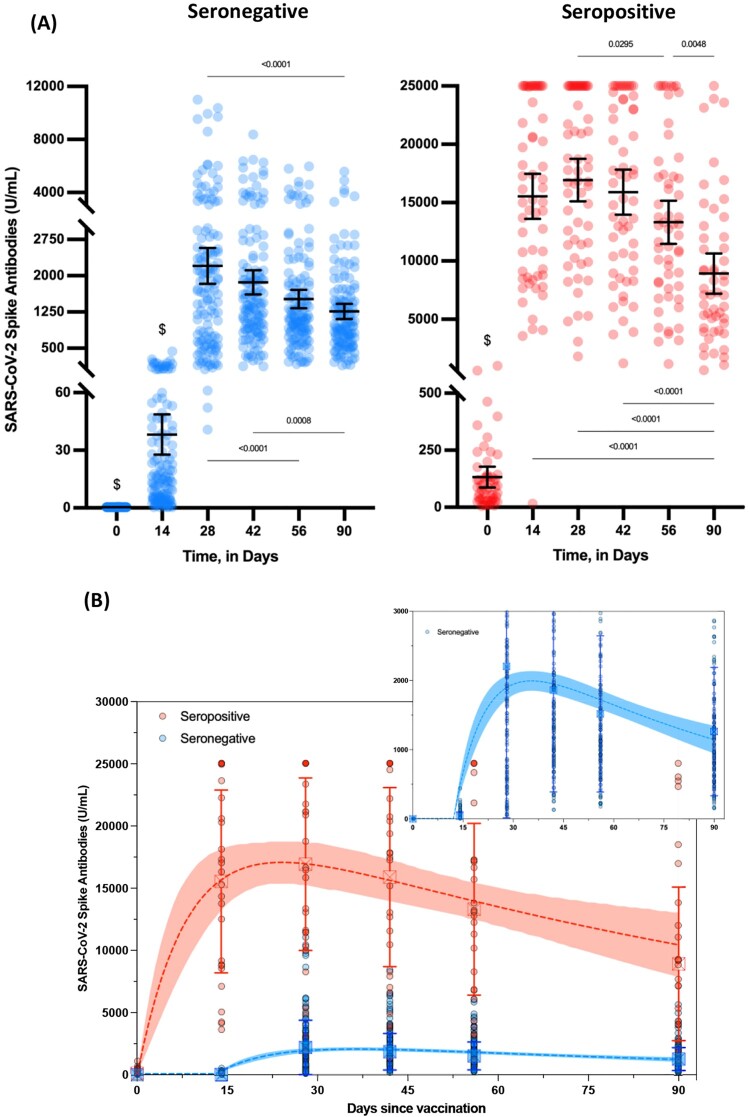
Evolution of SARS-CoV-2 spike antibodies (U/mL) in seronegative (blue) and seropositive individuals (red) according to the time since the first vaccine dose administration. (A) Means with 95% confidence intervals are shown. An automatic dilution of 1/100 at >250 U/mL was performed by the analyzer to extend the measurement domain up to 25,000 U/mL. Forty-six samples were rounded to 25,000 U/mL out of 1195 (3.8%). Results < 0.4 U/mL (limit of quantification) were rounded to 0.4. ^$^ = statistically different from all other groups (i.e. *P* < 0.0001). (B) Kinetic models of the humoral response based on a one-compartment model. A zoom of the seronegative population is presented in the right-upper part of the figure. Means with one standard deviation are shown.

In seronegative individuals, the rate of seroconversion 14 days after the first dose was 95.7% (Figure 2(A)). From day 28 to day 90, all participants had detectable anti-S antibodies. The maximal antibody response was reached between days 28 and 42 (2204 versus 1,863; *P* = 0.20), with a 48.8–57.7-fold increase compared to day 14 (i.e. 38.2 U/mL). Afterward, a continuous decrease was observed at days 56 (i.e. 1517 U/mL) and 90 (i.e. 1,262 U/mL) (Supplementary Table 1, Figure 2(A)). In seropositive individuals, the maximal antibody response was reached between days 14 and 42 (from 15,540 to 16,935; *P* >0.05), which represents a mean 122.1-fold increase compared to baseline (i.e. 132 U/mL). Afterward, a continuous decrease was observed at days 56 (i.e. 13,315 U/mL) and 90 (i.e. 8919 U/mL) (Supplementary Table 1, Figure 2(A)). All participants still had detectable anti-S antibodies up to day 90. Considering each time point separately, anti-S titres of seropositive individuals were always statistically higher compared to seronegative individuals (*P* < 0.0001) (Supplementary Table 1). Importantly, the inter-individual variability was important in each group.

The estimated half-life of antibodies observed from data collected until 90 days post-vaccination for seronegative participants was 55 days (95% CI: 37–107 days) as calculated by the one-compartmental model. The time to maximum concentration was estimated at 36 ± 3 days. In seropositive participants, the estimated half-life of antibodies after 90 days was 80 days (95% CI, 46–303 days) and the time to maximum concentration was 24 ± 4 days (Figure 2(B)).

## Discussion

In this study, we report a significant antibody decline 3 months post-vaccination in both seronegative and seropositive individuals who received two doses of the BNT162b2 vaccine. The highest mean antibody titre was observed between days 14 and 42 for seropositive participants and between 28 and 42 days for seronegative participants (Supplementary Table 1, Figure 2). Based on the one-compartment model, the time to maximum concentration was estimated at 24 ± 4 days in seropositive versus 36 ± 3 days in seronegative participants (Figure 2(B)). Previous studies also found an earlier maximal response in seropositive individuals [3,6].

At 3 months, a mean antibody decrease of 37.9% and 44.7% in seronegative and seropositive individuals was identified from the highest mean antibody response (Supplementary Table 1, Figure 2). Nevertheless, it is important to notice that all participants still had a robust antibody response at 3 months. Moreover, the vaccination with BNT162b2 elicited much higher antibody titres at 3 months compared to the titres collected in serum from convalescent patients using the same assay (i.e. Roche Elecsys anti-S pan-Ig assay) [8,9]. Using the half-lives derived from the kinetics model, we could predict a drop below the positivity threshold (i.e. 0.8 U/mL for anti-S) after 554 days for seronegative and after 1184 days for seropositive individuals. These predictions remain to date speculative and will need to be confirmed by subsequent sampling times but this could help to design vaccination strategies [10]. The aim is to keep sufficient antibody levels to protect vaccinated subjects against wild-type SARS-CoV-2 but also the related variants, which have all demonstrated some forms of immunity escape [10]. It has also been demonstrated that neutralizing antibodies better correlate with protection against infection than global serological testing and this may also serve in the future as a biomarker to ensure a proper protection at the patient’s level [11]. A high correlation (*r* > 0.86, *P* < 0.001) between the anti-S assay from Roche Diagnostics and a surrogate virus neutralization assay was found [12]. However, a limitation of this study included the lack of measurement of neutralizing capacity measurement. Data about the contribution of the cellular immune response are also missing.

Data about the long-term antibody kinetics in vaccinated subjects are still scarce. In a population of 33 healthy adults having received the Moderna mRNA-1273 vaccine and followed up to day 209, the estimated half-live was 52 days (95% CI: 46–58 days) using an exponential decay model [13]. In a cohort of 188 unvaccinated COVID-19 patients (mostly not hospitalized: 174/188) who were followed for up to 8 months, the antibody half-life was 83 days (95% CI: 62–126 days) [14]. All these results were consistent with the results obtained in this study in both seronegative (i.e. 55 days, 95% CI: 37–107 days) and seropositive participants (i.e. 80 days, 95% CI: 46–303 days). Because the 95% CI are overlapping, we cannot conclude that half-lives are different between seropositive and seronegative participants. This study (EudraCT registration number: 2020-006149-21) has a planned follow-up of two years, with the next blood sampling campaign planned in July 2021. This will permit further refine the kinetics model and to provide a better estimate of the antibody response in both seropositive and seronegative individuals.

## Supplementary Material

Supplemental Material
