# Supplementary material for: Antibody titres decline 3-month post-vaccination with BNT162b2
Source: Emerg Microbes Infect. 2021 Jul 22;10(1):1495–8. doi: 10.1080/22221751.2021.1953403 (PMC8300930; doi:10.1080/22221751.2021.1953403)
Supplement: Supplemental Material [file TEMI_A_1953403_SM3745.docx]

**Supplementary Table 1:** Evolution of SARS-CoV-2 spike antibodies (U/mL) in seronegative and seropositive persons. Means with 95% confidence intervals are shown. The between group difference of antibody titers were tested using a Tukey multiple comparison test. A multiple testing correction was applied in the multiple group comparision. P-value < 0.05 was considered significant.

|  | **Seronegative** | **Seropositive** | **P-value** |
| --- | --- | --- | --- |
| Before first dose | 0.40 (0.39-0.41) | 132.0 (86.1-177.6) | <0.0001 |
| 14 days | 38.2 (27.7-48.6) | 15,540 (13,606-17,473) | <0.0001 |
| 28 days | 2,204 (1,883-2,575) | 16,935 (15,112-18,759) | <0.0001 |
| 42 days | 1,863 (1,613-2,113) | 15,896 (13,968-17,824) | <0.0001 |
| 56 days | 1,517 (1,326-1,708) | 13,315 (11,464-15,165) | <0.0001 |
| 90 days | 1,262 (1,104-1,420) | 8,919 (7,201-10,637) | <0.0001 |
